# Osteoporosis in a Woman With Multiple Sclerosis: A Case Report

**DOI:** 10.7759/cureus.59287

**Published:** 2024-04-29

**Authors:** Ifigenia Kostoglou-Athanasiou, Lambros Athanassiou, Panagiotis Athanassiou, Andreas Giannakopoulos, Yehuda Shoenfeld

**Affiliations:** 1 Department of Endocrinology, Diabetes and Metabolism, Asclepeion Hospital, Voula, Athens, GRC; 2 Department of Rheumatology, Asclepeion Hospital, Voula, Athens, GRC; 3 Department of Rheumatology, St. Paul's Hospital, Thessaloniki, GRC; 4 8th Department of Orthopedics, Asclepeion Hospital, Voula, Athens, GRC; 5 Department of Internal Medicine, Zabludowicz Center for Autoimmune Diseases, Sheba Medical Center, Reichman University, Herzliya, ISR

**Keywords:** multiple sclerosis, fall, periprosthetic fracture, inflammatory cytokines, vitamin d, hip fracture, osteoporosis

## Abstract

Multiple sclerosis is a systemic autoimmune disease characterized by demyelination of nerves within the central nervous system. The prevalence of the disease is increasing. Cases with varying severity are observed. Multiple sclerosis is accompanied by severe osteoporosis, which may lead to fractures and may compromise patient mobility. The aim was to describe the case of a patient with multiple sclerosis who developed severe osteoporosis with multiple fractures.

A female patient was diagnosed with multiple sclerosis at the age of 52. At the age of 63, she presented with a fracture of the right femur. She was treated surgically with total arthroplasty. Osteoporosis was diagnosed and treatment was initiated. Seven months later the patient fell upon the fractured leg and developed a periprosthetic femoral fracture. She was treated with open reduction and internal fixation. Thereafter, bisphosphonates were administered. The patient can now walk with difficulty, independently, without orthotic help. In this case report, we have presented a case of multiple sclerosis who developed severe osteoporosis with multiple fractures.

## Introduction

Multiple sclerosis is a systemic autoimmune disease that results in demyelination of central nervous system neurons [1,2]. The disease has a variable course and may run either a rather benign course or a progressing course leading to disability [3]. Multiple sclerosis may be associated with osteoporosis, which may lead to fractures [4]. Multiple sclerosis is treated with immunomodulatory agents [5], including corticosteroids, which may affect bone metabolism [6]. In addition, the disease itself is an autoimmune disease [7,8]. Autoimmune diseases are accompanied by the release of inflammatory cytokines [8], which induce osteoclastogenesis [9,10], affect bone metabolism, and may induce osteoporosis [11]. Multiple sclerosis may be associated with vitamin D deficiency [12]. Vitamin D deficiency may induce osteomalacia and secondary osteoporosis. Multiple sclerosis causes mobility limitation, which may also affect bone metabolism and may induce muscular dysfunction, which affects bone metabolism. Multiple sclerosis is also accompanied by dysautonomia or dysregulation of the autonomic nervous system. Dysautonomia may cause osteoporosis via multiple mechanisms, both by affecting patient mobility as well as via its effects on bone metabolism. The prevalence of multiple sclerosis is 43.95 per 100.000 population and among those, 25% may have low bone mass and more than 10% may report a history of fracture [13].

Multiple sclerosis impairs gait and balance and is associated with falls [14,15]. Falls are a risk factor for fractures [16]. The aim was to describe the case of a patient with multiple sclerosis who developed severe osteoporosis and multiple fractures.

## Case presentation

**Figure 1:**
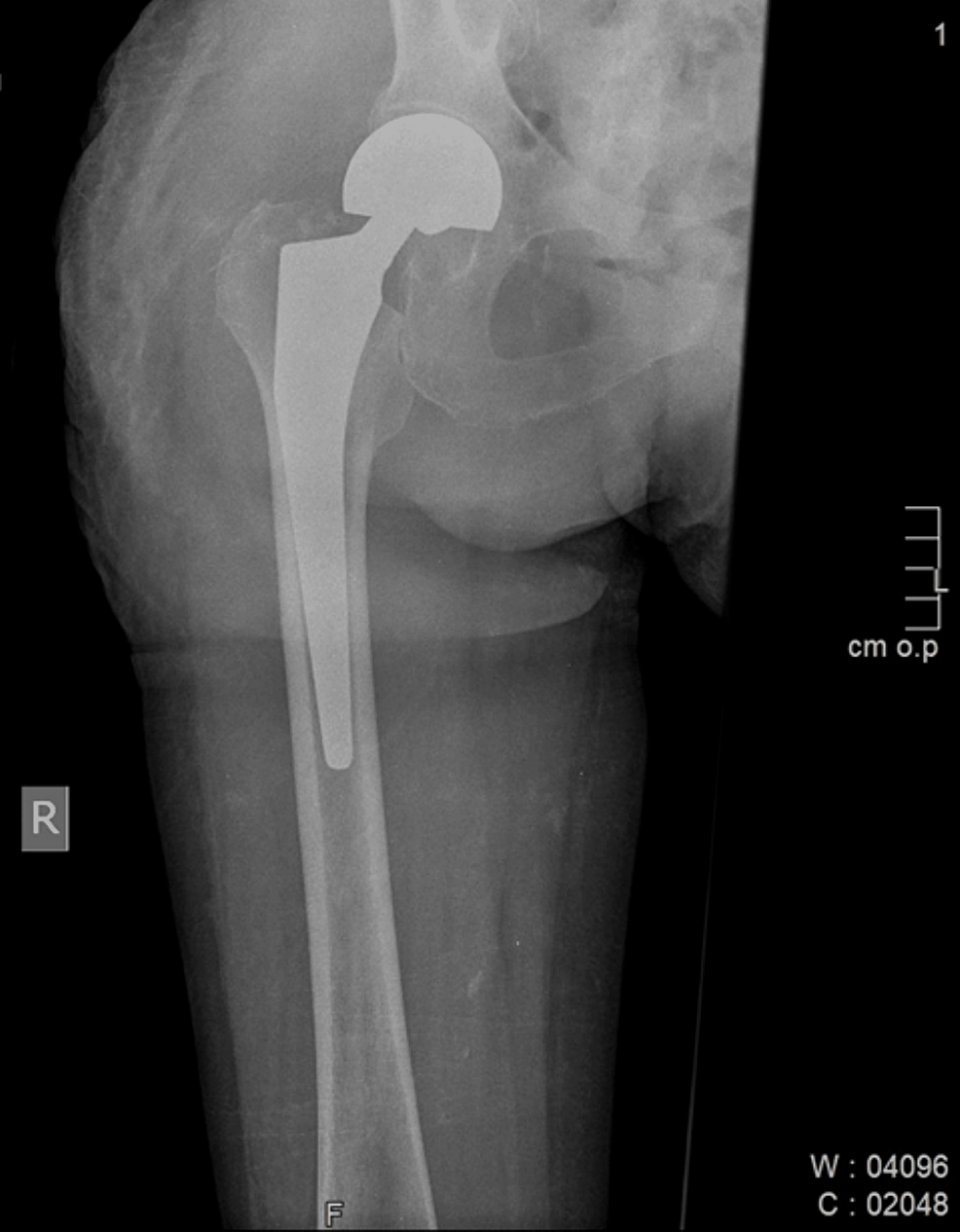
Hip fracture treated by total arthroplasty

This case is of a female patient who developed multiple sclerosis at the age of 51. She was treated by the administration of interferon beta-1-a. At the age of 58, she fell from a standing height, and she had a fracture of the right hip. She was treated surgically (Table 1). A total arthroplasty was performed (Figure 1). Thereafter, bone mineral density was measured and a T score in the left hip of -3.1 was observed. The patient improved and normal mobility was restored.

**Table 1:** Sequence of disease events in a female patient with multiple sclerosis.

Age	Disease event	Treatment
51 years	Diagnosis of multiple sclerosis	Interferon beta-1-a
58 years	Fall and right hip fracture	Arthroplasty
58 years and 7 months	Fall and periprosthetic fracture	Open reduction and internal fixation

An evaluation was performed to rule out any secondary cause of osteoporosis. Parathyroid hormone (PTH) was 31 pg/mL (normal range 15-65 pg/mL), serum urea 47 mg/dL (10-50 mg/dL), serum creatinine 0.7 mg/dL (0.5-1.5 mg/dL), serum calcium 9.1 mg/dL (8.5-10.5 mg/dL), serum phosphorus 3.2 mg/dL (2.5-4.5 mg/dL), serum alanine aminotransferase 36 U/L (8-45 U/L), serum aspartate aminotransferase 30 U/L (8-40 U/L), and 24 h urinary calcium levels were 150 mg/24h (20-275 mg/24h). The level of 25(OH)D3 was 31 ng/mL. Osteoporosis was diagnosed and denosumab was administered along with vitamin D and calcium.

**Figure 2:**
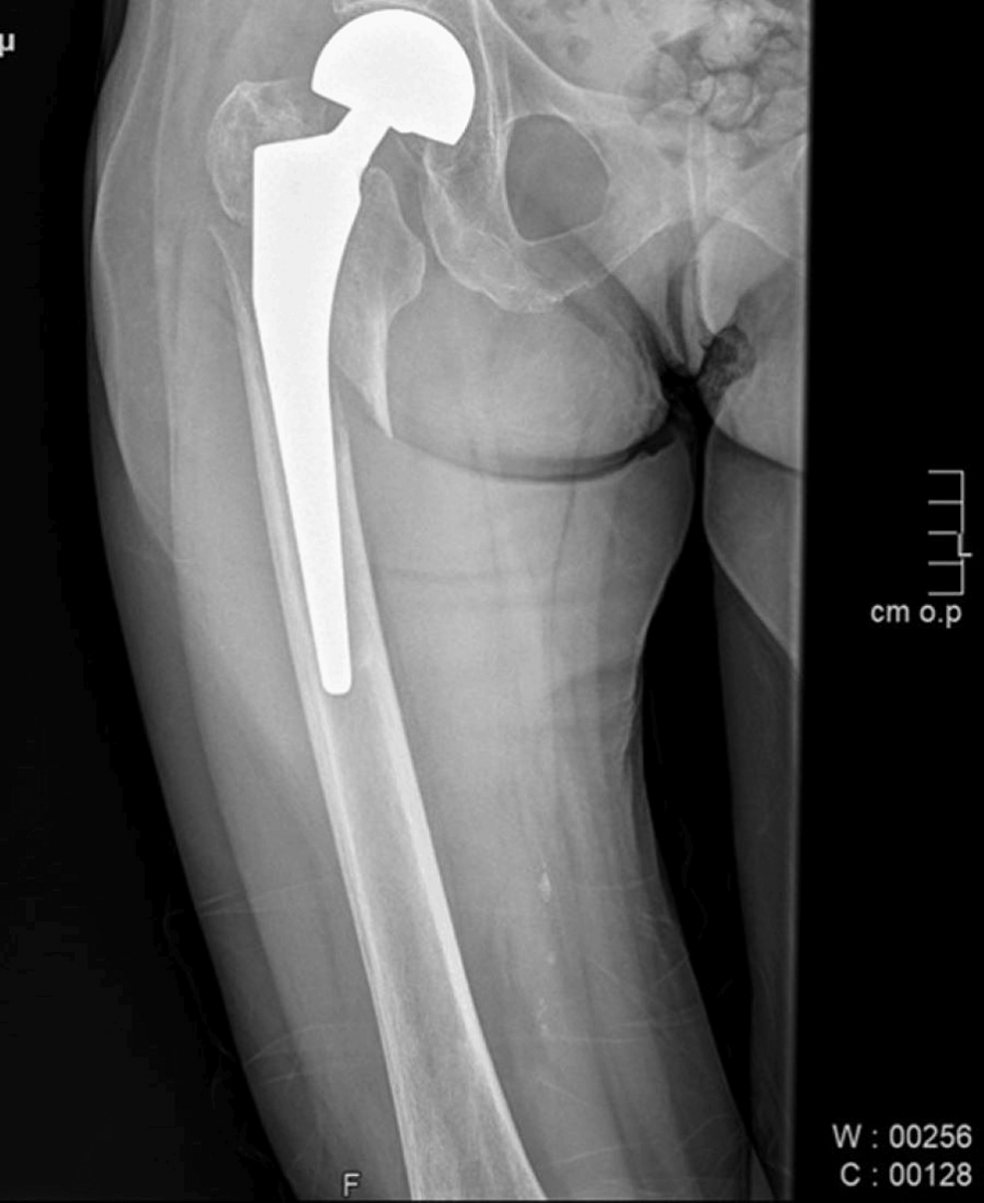
Periprosthetic femoral fracture

**Figure 3:**
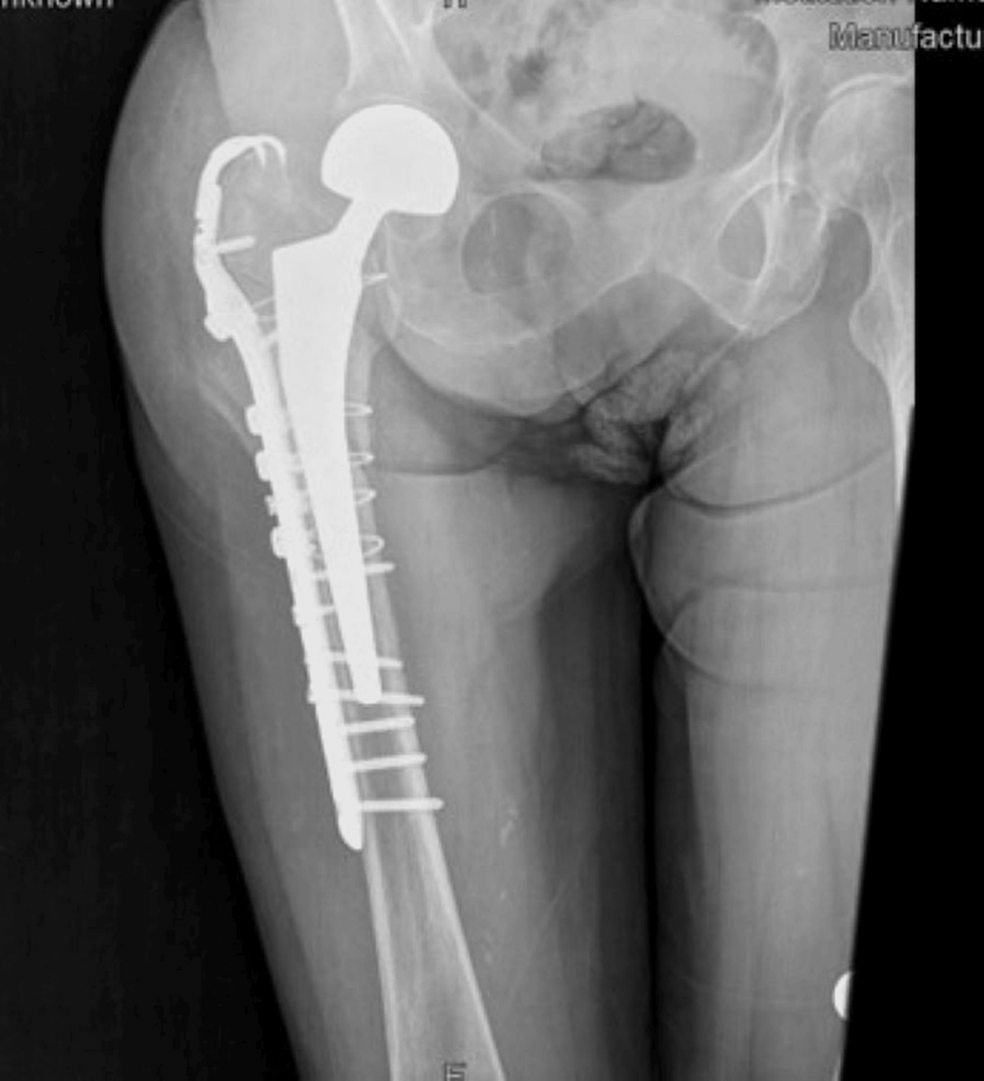
Periprosthetic femoral fracture treated by open reduction and internal fixation

Seven months after the first fracture, the patient fell again on the leg that had already been operated on. She developed a periprosthetic fracture (Figure 2). She was treated surgically by an open reduction and internal fixation (Figure 3). The patient can now walk with difficulty without using walking aids.

Bone mineral density was measured one month after the second operation and a T score of -3.1 was observed. Alendronate in the effervescent formulation was administered along with vitamin D and calcium. Following 12 months of treatment with alendronate, bone mineral density was re-evaluated, and a T score of -2.9 was observed in the left hip.

The patient was evaluated recently and her mobility was found improved.

## Discussion

Multiple sclerosis is a systemic inflammatory autoimmune disease that results in demyelination of neurons in the central nervous system [17]. The disease affects all organ systems and may impair patient mobility [14]. The disease is important to recognize, as it appears to have an increasing incidence and prevalence worldwide [18]. It is an example of an autoimmune disease with increasing incidence and prevalence along with systemic lupus erythematosus and diabetes mellitus type 1, which also have increased worldwide incidence and prevalence.

Multiple sclerosis is accompanied by an increased risk of osteoporosis, which may lead to fractures [4]. Multiple sclerosis causes demyelination, inflammation, and neuronal axonal damage, which leads to disruption of neuronal function. Disrupted neuronal function causes muscle weakness, sensory function impairment, and impairment of balance and vision. Multiple sclerosis leads to reduced bone mass and osteoporosis. Kampman et al. have argued that multiple sclerosis is a cause of secondary osteoporosis [19]. Various factors lead to osteoporosis. Reduced physical activity and limited exposure to mechanical load of the bones contribute to osteoporosis. Vitamin D deficiency may also be implicated in the pathogenesis of osteoporosis in the context of multiple sclerosis. Vitamin D deficiency has been observed in multiple sclerosis and may be implicated in the disease pathogenesis [12]. However, in the case described herein, the patient was on treatment with cholecalciferol and had normal vitamin D levels throughout her disease course. The use of immunomodulatory agents, corticosteroids in particular [6], may also be implicated in the pathogenesis of osteoporosis. However, in the case described herein, the patient had not received corticosteroids. She had received interferon beta 1-a. The use of interferon beta 1-a has not been found to be associated with osteoporosis. By contrast, it has been hypothesized that treatment with interferon beta 1-a may have beneficial effects on bone metabolism and may improve osteoporosis in the context of multiple sclerosis. In particular, it has been suggested that interferon treatment may inhibit osteoclastogenesis.

The disease is characterized by an autoimmune process involving the circulation of various inflammatory cytokines that affect bone metabolism [9]. In particular, circulating TNF-a may induce osteoclastogenesis [10]. Increased circulating levels of the inflammatory cytokine interleukin-6 (IL-6) have been observed in multiple sclerosis [8]. Il-6 induces osteoclastogenesis and may be involved in the pathogenesis of osteoporosis in patients with multiple sclerosis [9]. The interplay between autoimmunity and osteoporosis has been described extensively in the new field of osteoimmunology [20].

Dysautonomia, i.e., the dysregulation of the autonomic nervous system is observed in multiple sclerosis and may affect bone metabolism and induce osteoporosis in multiple ways [4], in particular by dysregulating the beneficial effects of the sympathetic and parasympathetic nervous system on bone physiology.

Multiple sclerosis impairs patient mobility and is a risk factor for falls [14,15]. Multiple sclerosis affects cognition, vision, balance, and gait thereby increasing the risk for falls. Over 50% of multiple sclerosis patients fall at least once a year [14]. Falls are a risk factor for fractures [16]. In the case of the patient described herein, a fall led to a hip fracture and in the course of the following year, another fall led to a periprosthetic fracture. The use of gait assistive devices, exercises, balance training, and home modifications have been discussed for the prevention of falls in multiple sclerosis patients.

In the case described herein, the patient developed a periprosthetic femoral fracture, which was treated surgically by open reduction, i.e., by the surgical realignment of bone fragments and internal fixation. Periprosthetic fractures are known to occur in the context of a pre-existing medical condition, such as multiple sclerosis, as in the case described.

Thus, multiple sclerosis may be associated with osteoporosis and fractures. A system has been developed for the assessment of the risk for the development of osteoporosis and fractures in multiple sclerosis patients [4], which takes into account traditional risk factors as well as the use of various drugs such as antidepressants and anticonvulsants and the history of falls. In this system, factors taken into account for the assessment of risk fracture in multiple sclerosis patients are female sex, age, the use of antidepressants, the use of anticonvulsants, history of falls, any previous fracture, the presence of fatigue, and smoking. According to this system, the patient described herein had a high risk for fracture. Osteoporosis is a multifactorial disease and should be treated in a multidimensional way, including modification of the way of life, involving cessation of smoking and alcohol and augmentation of physical activity. All the medications applied in the treatment of osteoporosis may be used in patients with multiple sclerosis. In the case described, denosumab and the effervescent formulation of alendronate were administered sequentially along with supplementation with calcium and vitamin D.

## Conclusions

Multiple sclerosis may be accompanied by osteoporosis. A propensity to falls is also observed in patients with multiple sclerosis. Osteoporosis and falls may lead to fractures. Fractures may need surgical management and may further compromise patient mobility in multiple sclerosis. Periprosthetic fractures may occur due to the propensity to falls. Thus, osteoporosis appears to be a dangerous comorbidity in multiple sclerosis. Patients with multiple sclerosis should be screened for osteoporosis and treated adequately to prevent falls and fractures.
